# Comparative Efficacy of Inhaled and Intravenous Corticosteroids in Managing COVID-19-Related Acute Respiratory Distress Syndrome

**DOI:** 10.3390/pharmaceutics16070952

**Published:** 2024-07-18

**Authors:** Ahmed A. Abdelkader, Bshra A. Alsfouk, Asmaa Saleh, Mohamed E. A. Abdelrahim, Haitham Saeed

**Affiliations:** 1 Clinical Pharmacy Department, Faculty of Pharmacy, Heliopolis University, Cairo 11765, Egypt; 2 Department of Pharmaceutical Sciences, College of Pharmacy, Princess Nourah bint Abdulrahman University, Riyadh 11671, Saudi Arabia; baalsfouk@pnu.edu.sa (B.A.A.); asali@pnu.edu.sa (A.S.); 3 Clinical Pharmacy Department, Faculty of Pharmacy, Beni-Suef University, Beni-Suef 62511, Egypt; mohamedemam9@yahoo.com (M.E.A.A.); haitham.sd1@gmail.com (H.S.)

**Keywords:** corticosteroids, COVID-19, dexamethasone, methylprednisolone, budesonide

## Abstract

Acute respiratory distress syndrome (ARDS) is a life-threatening condition in which the lungs fail to provide sufficient oxygen to the body’s vital organs. It is commonly associated with COVID-19 patients. Severe cases of COVID-19 can lead to lung damage and organ failure due to an immune response in the body. To mitigate these effects, corticosteroids, which are known for their anti-inflammatory properties, have been suggested as a potential treatment option. The primary focus of this study was to assess the impact of various corticosteroid administration methods on the outcomes of patients with COVID-19. Methods: The current study was conducted on COVID-19 patients divided into three groups. The first group was administered 6 mg of intravenous (IV) dexamethasone; the second group received 1 mg/kg of IV methylprednisolone (methylprednisolone); and the third group received budesonide respirable solution at a dosage of 1mg twice daily. The neubilizer used was a vibrating mesh nebulizer (VMN). All patients received standard care. We found that dexamethasone administered intravenously led to a significant reduction in C-reactive protein levels, surpassing the effectiveness of both IV methylprednisolone and inhaled budesonide. Oxygen saturation without mask change over time showed statistically significant differences (*p* = 0.004) in favor of the budesonide and dexamethasone groups for all days. Individuals who received methylprednisolone showed a significant decrease in mortality rate and an extended survival duration, with statistical significance observed at *p* = 0.024. The rest of the parameters, including ferritin, lymphocytes, total leukocyte count, platelets, hemoglobin, urea, serum potassium, serum sodium, serum creatinine, serum glutamic-pyruvic transaminase, serum glutamic-oxaloacetic transaminase, uric acid, albumin, globulin, erythrocyte sedimentation rate, international normalized ratio, oxygen saturation with flow, and oxygen flow, showed no statistically significant differences between the three drugs. In conclusion, treatment with IV methylprednisolone (1 mg/kg) resulted in a shorter hospital stay, decreased reliance on ventilation, and improved health outcomes for COVID-19 patients compared to using dexamethasone at a daily dosage of 6 mg or budesonide respirable solution at a dosage of 1mg twice daily.

## 1. Introduction

Acute respiratory distress syndrome (ARDS), commonly known as acute respiratory distress syndrome, is a life-threatening lung injury. It can be precipitated by sepsis, pneumonia, or even COVID-19. The onset of ARDS often occurs within hours to a few days of the underlying trauma, and its progression can be extremely rapid. Individuals suffering from ARDS may require admission to an intensive care unit (ICU) for ventilator support [1,2,3].

In December 2019, infection with an atypical form of pneumonia in a large population was reported in Wuhan City, China [4]. The suspected microorganism causing the widespread cases of pneumonia was discovered as a coronavirus, and then it was named severe acute respiratory syndrome type 2 (SARS-CoV-2), causing a disease called Coronavirus Disease 2019 (COVID-19) [5]. On 11 March 2020, the World Health Organisation (WHO) described the disease as an epidemic. By 2022, the COVID-19 pandemic had affected almost all of the world, and numerous waves of the infection had continued, leaving massive effects on countries’ health, economies, and political systems [6].

COVID-19 is an illness caused by a virus belonging to the SARS-CoV-2 family. It mainly impacts the lungs, leading to inflammation and pneumonia. Medical professionals classify the disease into three categories based on radiological factors: mild, moderate, and severe [7]. In mild cases, patients can be managed at home with symptomatic treatment. In a moderate-disease case, the patient requires hospital admission and is given supplemental oxygen, along with other necessary treatment modalities [8,9]. Patients with asymptomatic/presymptomatic and mild COVID-19 infection experience a range of symptoms, including fever, sore throat, cough, loss of taste and smell, headache, muscle pain, malaise, nausea, vomiting, and diarrhea [10].

Previous research on SARS indicated that the main cause of organ dysfunction was a disruption in regulation. Therefore, when the condition of COVID-19 patients worsens, there is an opportunity for intervention. During this period, corticosteroids and other immunosuppressive drugs can be beneficial, as they have been effective in treating SARS and MERS [11].

Severe COVID-19 is a form of viral pneumonia induced by infection with the SARS-CoV-2 coronavirus leading to ARDS. Its symptoms include a combination of viral pneumonia and ARDS [1,3,7,12].

As a result, the severity of clinical symptoms may be significantly linked to the inflammatory condition of COVID-19 patients. Patients with COVID-19 may experience an inflammatory response that can result in lung damage and multiple organ failure [13,14]. The potential ability of corticosteroids to reduce inflammation has been suggested as a strategy to prevent or mitigate these effects [15].

COVID-19 causes changes in the lungs that are similar to those seen in ARDS, including the destruction of the alveoli. Damage to the alveoli in the lungs occurs as a result of ARDS. During this phase, hyaline membranes form in the air sacs, followed by the swelling of tissues between cells. During the healing phase, there is an increase in growth [16]. As individuals progress through their condition, the long-term implications of COVID-19-related ARDS are being documented [17].

Corticosteroids have traditionally been used to treat respiratory diseases such as asthma, COPD (chronic obstructive pulmonary disease), severe bacterial pneumonia, and ARDS. Some recent studies have proposed the idea of utilizing short-term therapy with low doses of glucocorticoids for patients experiencing severe disease progression [18,19]. Certain studies have indicated that steroids can yield results by decreasing inflammation [20]. However, corticosteroids are immunosuppressive and can make patients more susceptible to superadded infection [21].

Corticosteroids, such as dexamethasone, prednisone, methylprednisolone, and hydrocortisone, have been utilized in several clinical studies to treat COVID-19 patients with mild, moderate, severe, and critical illness [10]. Most individuals who contract COVID-19 die as a result of a response triggered by the infection rather than the disease itself [22]. Corticosteroids play a role in controlling the cytokine storm, helping patients with lung damage caused by an overactive immune response, as seen in severe cases of COVID-19. However, corticosteroids have been shown to have effects such as prolonging the time needed for the virus to clear and increasing the risk of infection. They do, however, reduce the duration of ventilation time spent in the ICU and, most importantly, the mortality rate among COVID-19 patients [23].

Corticosteroids can be administered through various methods. For instance, systemic steroids are commonly used to treat various immune-related conditions. However, it is crucial to manage the dosage to avoid one of their known side effects: suppression of the hypothalamic–pituitary–adrenal (HPA) axis. On the other hand, when localized treatment is needed for conditions like obstructive pulmonary disease (COPD) or asthma, corticosteroids can be applied topically or inhaled. The two primary approaches investigated for COVID-19 treatment have been inhalation and systemic administration.

The current study aims to evaluate the impact of different delivery routes of corticosteroids on the outcomes of COVID-19 patients. Three different corticosteroids (dexamethasone, methylprednisolone and budesonide) were compared to determine their effects on different patient parameters as ferritin, lymphocytes, total leukocyte count, platelets, hemoglobin, urea, serum potassium, serum sodium, serum creatinine, serum glutamic-pyruvic transaminase, serum glutamic-oxaloacetic transaminase, uric acid, albumin, globulin, erythrocyte sedimentation rate, international normalized ratio, oxygen saturation and mortality rate of those hospitalized in the intensive care unit.

## 2. Materials and Methods

Patients who were diagnosed according to WHO criteria guidelines and those who were admitted to the hospital and tested positive for SARS-CoV-2 infection using real-time PCR were included if they were over 18 years old.

Criteria for inclusion: This study focused on patients with severe cases of COVID-19 who tested positive for PCR. The study included patients whose oxygen saturation levels dropped below 94%, PaO_2_/FiO_2_ < 300 mm Hg, a respiratory rate > 30 breaths/min, or lung infiltrates > 50%. These patients may experience rapid clinical deterioration and should be given oxygen therapy and be hospitalized. Additionally, patients were required to provide consent [24].

Criteria for exclusion: Individuals with diabetes mellitus (DM) or hypertension, those with contraindications for receiving steroids, individuals with immunodeficiency disorders, and those with oxygen saturation levels above 94% on room air were excluded from the study, as well as mechanically ventilated patients. Additionally, individuals who did not indicate a willingness to participate in the research were excluded [25]. The patients were followed up until they were discharged from the hospital or until death. Three groups of patients were formed. The first and second groups each started with 38 patients, while the third group started with 34 patients. Patients who continued the study were 31, 28, and 26 for the 1st, 2nd, and 3rd group, respectively. The first group received dexamethasone, the second group received methylprednisolone, and the third group received a budesonide inhaled solution. A total of 25 patients did not continue the study according to the following causes: 8 patients refused to sign the informed consent, 11 patients were transferred to other hospitals, and 6 patients decided not to continue the study. Patients were recruited in groups randomly using a computer-based randomizer (www.randomizer.org, accessed on 10 January 2021).

The first group received a dose of 6 mg of dexamethasone (Amriya, Cairo, Egypt). Meanwhile, the second group received a dose of 1mg/kg/day of methylprednisolone (Solu-Medrol, Pfizer, Cairo, Egypt) administered over 60 min. The 3rd group received a budesonide inhaled solution at a dosage of 1 mg/twice daily (Pulmicort, AstraZeneca, Cairo, Egypt). Those doses are equivalent according to guidelines [26].

Inhaled corticosteroids were administered to patients using a vibrating mesh nebulizer (Aerogen Solo, Aerogen Limited, Galway, Ireland). The nebulizer was attached to a T-piece, which was configured with a face mask on one side and a filter on the other. This setup facilitated the effective delivery of corticosteroids directly to the patients’ respiratory tracts, ensuring optimal deposition of the medication in the lungs while reducing the risk of aerosol dispersion and contamination.

We carefully monitored factors such as information, existing medical conditions, smoking habits, oxygen levels, the type of oxygen support used, and respiratory rate. Furthermore, we recorded the utilization of a ventilator, the length of the hospital stay, and any instances of mortality. On the discharge date, a final assessment was conducted. In addition, routine follow up for all patients included monitoring fever, symptoms, glycemic parameters, blood pressure, and complete blood picture.

The device used for measuring samples was QuantStudio 5 Real-Time PCR System (Thermo Fisher Scientific Inc., Waltham, MA, USA), which was utilized for PCR testing, providing quantification of viral RNA. Additionally, a Mindray BC 20s (Mindray, Shenzhen, China) was employed for hematological analyses, offering comprehensive blood cell counts and diagnostics essential for understanding the hematological impact of COVID-19. Furthermore, a ST-200 CC Blood Gas Analyzer (Wondfo, Guangzhou, China) was used to measure blood gas parameters.

Studying the imagistic characteristics of ARDS before and after treatment gives vital information on the changing physiological processes and effectiveness of therapy in affected patients. Prior to treatment, imaging techniques such as chest radiography and computed tomography (CT) scans displayed characteristic attributes such as bilateral opacities, ground-glass opacities, and consolidation, which indicate serious damage to the air sacs in the lungs and hindered exchange of gases. These images are essential diagnostic tools that assist in promptly identifying and assessing the severity of a condition. After therapeutic measures, imaging is crucial for assessing the response to treatment and the course of the disease. Resolution of opacities, reduction in pulmonary infiltrates, and improvement in aeration are signs that the treatment is effective and the patient is recovering.

The main outcomes focused on the length of patients’ hospital stay and the occurrence of any deaths during their stay. Additionally, we examined factors such as CRP levels and the date patients were discharged after enrollment.

We also investigated outcomes such as the need for mechanical ventilation and admission to the intensive care unit (ICU). We assessed the percentage of patients who experienced these outcomes at the time of discharge.

Soon after the patient was admitted, various laboratory tests were conducted, including assessments for kidney and liver function, arterial blood gases, lactate dehydrogenase levels, D-dimer tests, serum ferritin analysis, and C-reactive protein screening. To prevent thromboembolism during their hospital stay, all patients were administered low-molecular-weight heparins.

The criteria for improvement focused on the duration of the patient’s significant recovery, which determined their readiness for discharge. This included reducing the need for oxygen support, such as by transitioning from methods like non-rebreathing masks or mechanical ventilation to using nasal cannulas. Other factors considered included ICU transfers, mortality rates, and readmission within 30 days after discharge from the hospital due to infection.

Changes in the patient’s radiological and biochemical parameters were observed on both the admission day (day 1) and day 6 after administering steroids (methylprednisolone, dexamethasone, or budesonide) for 11 days [27]. Additionally, we examined the correlation between steroid usage, CRP levels, and the presence of infiltrates on chest imaging reports of patients. It was found that the administration of corticosteroids led to improvements in both CRP levels and X-ray results [9]. In this study, we compared the outcomes among patients treated with methylprednisolone, dexamethasone, or budesonide using this approach.

Informed Consent: Each patient provided consent before participating in the study. The research was conducted following the guidelines outlined in the Declaration of Helsinki. The study was approved by the Ethics Committee of Beni-sueif University (EC H PhBSU 21007) for studies involving human subjects.

Statistical analysis is carried out by Way of Analysis of Variance (ANOVA). Comparison between the study groups was performed using an ANOVA test with Bonferroni post hoc multiple 2-group comparisons.

## 3. Results

This study evaluated the effectiveness of inhaled budesonide, intravenous (IV) dexamethasone, and IV methylprednisolone in treating 85 patients with COVID-19-related ARDS across two distinct phases: early treatment (days 1–6) and later treatment (days 6–11). Key findings from the study are summarized below, as shown in Table 1 and Table 2.

Early Treatment Phase (Days 1–6): During the initial treatment phase, IV dexamethasone demonstrated a statistically significant improvement in key inflammatory and respiratory parameters compared to inhaled budesonide. Specifically, reductions in C-reactive protein (CRP) and enhancements in hemoglobin (HB) levels and oxygen saturation were noted, suggesting a more potent anti-inflammatory effect (*p* < 0.05). Conversely, IV methylprednisolone showed a distinct advantage over IV dexamethasone in the management of serum potassium levels, indicating a possible benefit in electrolyte stabilization (*p* < 0.05). Additionally, inhaled budesonide was superior to IV methylprednisolone in improving oxygen levels, providing evidence of its efficacy in direct respiratory management without the systemic side effects associated with IV administration (*p* < 0.05). Other parameters such as ferritin, lymphocytes, total leukocyte count (TLC), platelets, serum creatinine, serum sodium, liver enzymes (SGPT, SGOT), uric acid, erythrocyte sedimentation rate (ESR), international normalized ratio (INR), and further measures of oxygen saturation did not show significant differences, suggesting comparable effects among the treatments in these aspects (*p* > 0.05).

Later Treatment Phase (Days 6–11): In the later phase of treatment, IV dexamethasone continued to exhibit superior performance in controlling additional parameters such as urea, serum sodium, uric acid, and INR when compared to IV methylprednisolone, alongside better oxygen saturation relative to inhaled budesonide (*p* < 0.05). This reinforces the role of IV dexamethasone in managing the broader systemic effects of severe ARDS. In contrast, inhaled budesonide showed a significant advantage over IV methylprednisolone in terms of serum sodium and oxygen saturation improvements (*p* < 0.05), highlighting its potential for managing specific systemic and respiratory challenges effectively. Similar to the initial treatment phase, no significant differences were observed in the remaining parameters including CRP, ferritin, lymphocytes, TLC, platelets, HB, serum creatinine, and liver enzymes, indicating that the three drugs have a generally comparable impact on these metrics (*p* > 0.05).

**Figure 1:**
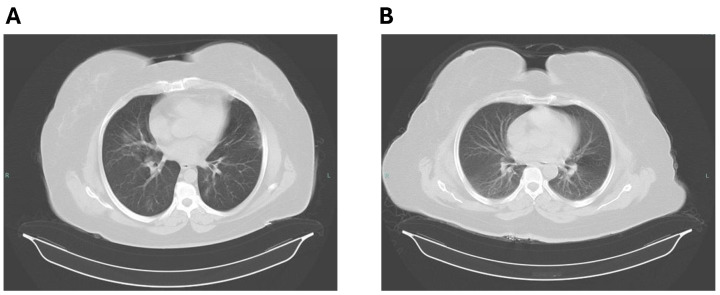
Computed tomography scan showing bilateral scattered patches of faint ground glass opacity (**A**) before and after treatment initiation (**B**).

Enhancement of imagistic findings was considered for evaluation of clinical status of patients, as shown in Figure 1.

Mortality and Hospital Stay: Importantly, there were no statistically significant differences in mortality rates or the duration of hospital stays among the three treatment groups throughout the study period (*p* > 0.05). This suggests that while there are differences in the biochemical response to these treatments, these variations do not translate into differences in these critical outcomes.

## 4. Discussion

The results of this clinical study provide valuable insights into the comparative efficacy of inhaled budesonide, intravenous (IV) dexamethasone, and IV methylprednisolone in managing COVID-19-related acute respiratory distress syndrome (ARDS). Each treatment modality presents distinct advantages and limitations that warrant consideration in clinical practice, particularly in the management of severe respiratory complications associated with COVID-19.

COVID-19 has a negative impact on many patients with other comorbidities. Recent literature underscores the profound repercussions of the COVID-19 pandemic on patients with hepatocellular carcinoma (HCC). The study illuminates a stark reality: delays in screening programs, diagnosis, and treatment initiation have significantly exacerbated the clinical outcomes for individuals already burdened with HCC [28]. As healthcare systems globally pivoted resources to combat COVID-19, essential oncological services experienced unprecedented disruptions, leading to missed opportunities for early detection and timely interventions in HCC cases [29,30]. These findings underscore the urgent need for strategic healthcare policies aimed at mitigating the collateral damage of future health crises on vulnerable patient populations, emphasizing the imperative of maintaining uninterrupted access to critical cancer care services.

Regarding the differential efficacy of different treatment options, IV dexamethasone exhibited superior control over systemic inflammation and respiratory function, as evidenced by significant improvements in C-reactive protein (CRP), hemoglobin (HB), and oxygen saturation. This aligns with the known potent anti-inflammatory effects of dexamethasone, which can be crucial in mitigating the cytokine storm associated with severe COVID-19 cases. An uncontrolled overproduction of soluble inflammatory markers, known as a “cytokine storm”, is primarily responsible for generating ARDS. The cytokine storm spreads swiftly, and the immune system begins to assault the body, leading to multiple organ failure and final death. CCL5 is an effective chemoattractant for B cells, monocytes, T cells, eosinophils, dendritic cells, and natural killer cells. CCL5 also causes basophils to release histamine and eosinophils to produce cationic proteins. Histamine stimulates an abnormal immunological response, resulting in cytokine storms and, eventually, multiple organ failure. A recent study found that dexamethasone binds strongly to CCL5 and creates a stable drug–ligand complex. The binding of unregulated CCL5 production in COVID-19 patients might minimize the risk of severe lung damage caused by inflammatory reactions, resulting in lower morbidity and mortality [31]. The finding that dexamethasone effectively reduces systemic inflammatory markers and improves oxygenation supports its use in severe ARDS, where systemic inflammation plays a key role in patient deterioration. A recent randomized controlled trial showed that early dexamethasone medication may shorten the duration of mechanical breathing and overall mortality in individuals with established moderate-to-severe ARDS. Another study by Hegeman et al. claimed that liposome-encapsulated dexamethasone given by IV injection attenuated ventilator-induced lung inflammation with minimal systemic side effects in a mouse model [32,33].

Conversely, inhaled budesonide demonstrated efficacy in directly targeting respiratory tissues while minimizing systemic exposure. This targeted approach resulted in significant improvements in oxygen saturation without the broader systemic impacts observed with IV corticosteroids. The benefits of inhaled corticosteroids in reducing potential systemic side effects while effectively managing localized pulmonary symptoms provide a compelling argument for their use, particularly in patients who may be at higher risk for steroid-related complications. The results of a recent phase 2 randomized clinical trial indicated that early treatment with inhaled budesonide in patients with COVID-19 might prevent clinical deterioration, as determined by a reduced number of urgent care visits. Another recent study highlighted that budesonide nebulization improved peak and plateau airway pressures, oxygenation, and reduced inflammatory cytokines (IL-1β, IL-6, TNF-α) in patients with early [34,35,36].

Regarding electrolyte management, the advantage of IV methylprednisolone in managing serum potassium levels highlights its potential utility in situations where electrolyte imbalances are a concern. Electrolyte management is crucial in critically ill patients, as imbalances can exacerbate the condition and lead to complications. The ability of methylprednisolone to stabilize potassium levels may be particularly beneficial in the subset of ARDS patients who also suffer from renal complications or those who are receiving other treatments that can affect electrolyte levels. A study showed that methylprednisolone infusion produced an average fall in plasma potassium from 3–99 to 3–10 mmol/l, which was associated with an increase in plasma glucose and serum insulin, suggesting that this arose from a shift of potassium from the extracellular to the intracellular space [37,38].

Multiple studies have found that methylprednisolone has an impact on the patient’s treatment progress and overall outcome. This includes improvements in status scores (measured using a scale), shorter hospital stays, and reduced reliance on mechanical ventilation. Furthermore, individuals who were administered methylprednisolone exhibited a higher mortality rate compared to those who received dexamethasone (8 versus 15) [27,39,40]. However, it is worth noting that this difference did not reach significance [41,42]. Given a larger sample size, it is conceivable that we would discover statistically meaningful variations [43,44].

In a study that looked back on a group of patients, we examined the effectiveness of using low-dose methylprednisolone for a period to treat COVID-19. We found that patients who received 1–2 mg/kg/day methylprednisolone for 5–7 days had shorter hospital stays and were less likely to require ventilation compared to those who received standard care. However, we did not observe any difference in mortality rates between the two groups, which is consistent with our findings [45]. Patients who received methylprednisolone showed a decrease in outcomes [46].

No Significant Differences in Some Parameters: It is noteworthy that many parameters, such as ferritin, lymphocytes, and other biochemical markers, did not differ significantly between treatment groups. This suggests that while specific treatments may offer advantages in certain areas, the overall biochemical impact of these corticosteroids might be more uniform than expected. This finding could indicate that the choice of corticosteroid might be less crucial for the general management of these parameters but more relevant to targeted outcomes like inflammation and oxygenation. Because of their diverse effects, corticosteroids have been used therapeutically for treating a wide variety of auto-immune, rheumatologic, inflammatory, neoplastic, and infectious diseases [47].

The lack of differences in mortality rates and hospital stays among the treatment groups is a critical finding. It implies that while individual drugs may influence biochemical markers and physiological parameters differently, these variations do not necessarily translate into different clinical outcomes such as survival or length of hospitalization. This could suggest that factors other than the choice of corticosteroids, such as underlying health conditions, the timing of intervention, or supportive care measures, play more decisive roles in determining the overall prognosis in ARDS patients. To dovetail with this, a recent study revealed that independent predictors of 100-day mortality in patients with ARDS include age (per log 10 years), BMI < 24, SOFA score, lymphocyte count, and lymphocyte/neutrophil ratio. Additionally, ARDS patients with a lymphocyte/neutrophil ratio < 0.0537 had a greater 28-day death rate compared to those with a ratio > 0.0537. The lymphocyte/neutrophil ratio was also found to be a robust and independent predictor of outcome in ARDS patients, particularly those with atypical immunodeficiency [48,49].

### Limitations

The study’s limitations include its short duration and the specific patient population, which may limit the generalizability of the findings. Additionally, as the study only compares three specific corticosteroid treatments, further research could explore other therapeutic agents or combinations thereof to ascertain the most effective and safe treatment protocols for different ARDS patient subgroups. Longitudinal studies could also help in understanding the long-term effects of these treatments on patient recovery and quality of life post-ARDS [50,51,52].

## 5. Conclusions

The study indicates that IV dexamethasone may offer superior benefits in managing certain biochemical parameters like CRP, HB, and oxygen saturation in COVID-19-related ARDS compared to inhaled budesonide. However, inhaled budesonide shows potential advantages in oxygen management without the systemic effects seen with IV corticosteroids. IV methylprednisolone’s performance varied across different measures, but it was occasionally superior in managing serum potassium levels. Overall, none of the treatment methods showed a significant impact on mortality rates or duration of hospital stay.

## Figures and Tables

**Table 1:** Laboratory findings for all groups at baseline, 6 days, and 11 days of hospital stay.

Parameter	(I) Group	(J) Group	*p*-Value	Parameter	(I) Group	(J) Group	*p*-Value
	Day 1 vs. Day 6				Day 1 vs. Day 11		
**D6-D1-CRP**	Dexamethasone	budesonide	0.012	**D11-D1-CRP**	Dexamethasone	budesonide	1.000
		Methylprednisolone	0.346			Methylprednisolone	0.394
	Budesonide	Methylprednisolone	0.346		Budesonide	Methylprednisolone	1.000
**D6-D1-Ferritin**	Dexamethasone	budesonide	1.000	**D11-D1-Ferritin**	Dexamethasone	budesonide	0.696
		Methylprednisolone	0.374			Methylprednisolone	0.268
	Budesonide	Methylprednisolone	1.000		Budesonide	Methylprednisolone	1.000
**D6-D1-Lympho**	Dexamethasone	budesonide	1.000	**D11-D1-Lympho**	Dexamethasone	budesonide	1.000
		Methylprednisolone	0.910			Methylprednisolone	1.000
	Budesonide	Methylprednisolone	0.298		Budesonide	Methylprednisolone	1.000
**D6-D1-TLC**	Dexamethasone	budesonide	1.000	**D11-D1-TLC**	Dexamethasone	budesonide	1.000
		Methylprednisolone	0.101			Methylprednisolone	0.075
	Budesonide	Methylprednisolone	1.000		Budesonide	Methylprednisolone	1.000
**D6-D1-PLTs**	Dexamethasone	budesonide	0.087	**D11-D1-PLTs**	Dexamethasone	budesonide	1.000
		Methylprednisolone	0.058			Methylprednisolone	0.042
	Budesonide	Methylprednisolone	1.000		Budesonide	Methylprednisolone	0.808
**D6-D1-Hb**	Dexamethasone	budesonide	0.613	**D11-D1-Hb**	Dexamethasone	budesonide	1.000
		Methylprednisolone	0.001			Methylprednisolone	0.081
	Budesonide	Methylprednisolone	0.199		Budesonide	Methylprednisolone	0.705
**D6-D1-Urea**	Dexamethasone	budesonide	1.000	**D11-D1-Urea**	Dexamethasone	budesonide	0.809
		Methylprednisolone	1.000			Methylprednisolone	1.000
	Budesonide	Methylprednisolone	1.000		Budesonide	Methylprednisolone	1.000
**D6-D1-s.K**	Dexamethasone	budesonide	0.615	**D11-D1-s.K**	Dexamethasone	budesonide	1.000
		Methylprednisolone	0.007			Methylprednisolone	0.167
	budesonide	Methylprednisolone	0.562		Budesonide	Methylprednisolone	1.000
**D6-D1-s.Creat**	Dexamethasone	budesonide	1.000	**D11-D1-s.Creat**	Dexamethasone	budesonide	1.000
		Methylprednisolone	0.549			Methylprednisolone	0.097
	Budesonide	Methylprednisolone	1.000		Budesonide	Methylprednisolone	0.261
**D6-D1-s.Na**	Dexamethasone	budesonide	0.379	**D11-D1-s.Na**	Dexamethasone	budesonide	0.906
		Methylprednisolone	0.098			Methylprednisolone	1.000
	Budesonide	Methylprednisolone	1.000		Budesonide	Methylprednisolone	0.523
**D6-D1-SGPT**	Dexamethasone	budesonide	1.000	**D11-D1-SGPT**	Dexamethasone	budesonide	1.000
		Methylprednisolone	0.873			Methylprednisolone	1.000
	Budesonide	Methylprednisolone	1.000		Budesonide	Methylprednisolone	1.000
**D6-D1-SGOT**	Dexamethasone	budesonide	0.715	**D11-D1-SGOT**	Dexamethasone	budesonide	1.000
		Methylprednisolone	0.295			Methylprednisolone	0.412
	Budesonide	Methylprednisolone	1.000		Budesonide	Methylprednisolone	1.000
**D6-D1-Uric acid**	Dexamethasone	budesonide	1.000	**D11-D1-Uric acid**	Dexamethasone	budesonide	1.000
		Methylprednisolone	1.000			Methylprednisolone	0.131
	Budesonide	Methylprednisolone	1.000		Budesonide	Methylprednisolone	0.284
**D6-D1-ESR**	Dexamethasone	budesonide	1.000	**D11-D1-ESR**	Dexamethasone	budesonide	0.961
		Methylprednisolone	0.616			Methylprednisolone	0.799
	Budesonide	Methylprednisolone	1.000		Budesonide	Methylprednisolone	1.000
**D6-D1-INR**	Dexamethasone	budesonide	0.921	**D11-D1-INR**	Dexamethasone	budesonide	1.000
		Methylprednisolone	1.000			Methylprednisolone	1.000
	Budesonide	Methylprednisolone	1.000		budesonide	Methylprednisolone	1.000
**D6-D1-O2 Sat without flow**	Dexamethasone	budesonide	0.700	**D11-D1-O2 Sat without flow**	Dexamethasone	budesonide	1.000
		Methylprednisolone	0.146			Methylprednisolone	0.337
	Budesonide	Methylprednisolone	1.000		Budesonide	Methylprednisolone	1.000
**D6-D1-O2 Sat with flow**	Dexamethasone	budesonide	0.000	**D11-D1-O2 Sat with flow**	Dexamethasone	budesonide	0.000
		Methylprednisolone	1.000			Methylprednisolone	1.000
	Budesonide	Methylprednisolone	0.000		Budesonide	Methylprednisolone	0.000

**Table 2:** Laboratory findings for all groups at 6 days, and 11 days of hospital stay.

Parameter	(I) Group	(J) Group	*p*-Value
	Day 1 vs. Day 6		
**D11-D6-CRP**	Dexamethasone	Budesonide	1.000
		Methylprednisolone	0.180
	Budesonide	Methylprednisolone	0.278
**D11-D6-Ferritin**	Dexamethasone	Budesonide	1.000
		Methylprednisolone	0.433
	Budesonide	Methylprednisolone	1.000
**D11-D6-Lympho**	Dexamethasone	Budesonide	1.000
		Methylprednisolone	0.070
	Budesonide	Methylprednisolone	0.795
**D11-D6-TLC**	Dexamethasone	Budesonide	1.000
		Methylprednisolone	1.000
	Budesonide	Methylprednisolone	1.000
**D11-D6-PLTs**	Dexamethasone	Budesonide	1.000
		Methylprednisolone	1.000
	Budesonide	Methylprednisolone	1.000
**D11-D6-Hb**	Dexamethasone	Budesonide	1.000
		Methylprednisolone	1.000
	Budesonide	Methylprednisolone	1.000
**D11-D6-Urea**	Dexamethasone	Budesonide	1.000
		Methylprednisolone	0.049
	Budesonide	Methylprednisolone	1.000
**D11-D6-s.K**	Dexamethasone	Budesonide	1.000
		Methylprednisolone	0.794
	Budesonide	Methylprednisolone	1.000
**D11-D6-s.Creat**	Dexamethasone	Budesonide	0.699
		Methylprednisolone	1.000
	Budesonide	Methylprednisolone	0.353
**D11-D6-s.Na**	Dexamethasone	Budesonide	0.978
		Methylprednisolone	0.000
	Budesonide	Methylprednisolone	0.008
**D11-D6-SGPT**	Dexamethasone	Budesonide	1.000
		Methylprednisolone	0.051
	Pulmicort	Methylprednisolone	0.149
**D11-D6-SGOT**	Dexamethasone	Budesonide	1.000
		Methylprednisolone	0.484
	Budesonide	Methylprednisolone	1.000
**D11-D6-Uric acid**	Dexamethasone	Budesonide	1.000
		Methylprednisolone	0.003
	Budesonide	Methylprednisolone	0.175
**D11-D6-ESR**	Dexamethasone	Budesonide	0.242
		Methylprednisolone	0.941
	Budesonide	Methylprednisolone	0.644
**D11-D6-INR**	Dexamethasone	Budesonide	1.000
		Methylprednisolone	0.022
	Budesonide	Methylprednisolone	0.075
**D11-D6-O2 Sat without flow**	Dexamethasone	Budesonide	1.000
		Methylprednisolone	0.283
	Budesonide	Methylprednisolone	1.000
**D6-D1-O2 Sat with flow**	Dexamethasone	Budesonide	0.000
		Methylprednisolone	1.000
	Budesonide	Methylprednisolone	0.000
**Hospital Stay**	Dexamethasone	Budesonide	1.000
		Methylprednisolone	0.948
	Budesonide	Methylprednisolone	1.000

## Data Availability

The data presented in this study are available on request from the corresponding author. The data are not publicly available due to privacy and confidentiality.
